# *In Ovo* Delivery of CpG DNA Reduces Avian Infectious Laryngotracheitis Virus Induced Mortality and Morbidity

**DOI:** 10.3390/v7041832

**Published:** 2015-04-08

**Authors:** Simrika Thapa, Mohamed Sarjoon Abdul Cader, Kalamathy Murugananthan, Eva Nagy, Shayan Sharif, Markus Czub, Mohamed Faizal Abdul-Careem

**Affiliations:** 1 Faculty of Veterinary Medicine, University of Calgary, Health Research Innovation Center 2C64, 3330 Hospital Drive NW, Calgary, AB T2N 4N1, Canada; E-Mails: sthapa@ucalgary.ca (S.T.); sarjoonhafeez@gmail.com (M.S.A.C.); kalamathy6@gmail.com (K.M.); mmczub@ucalgary.ca (M.C.); 2 Department of Pathobiology, University of Guelph, Guelph, ON N1G 2W1, Canada; E-Mails: enagy@ovc.uoguelph.ca (E.N.); shayan@uoguelph.ca (S.S.)

**Keywords:** *In ovo*, toll-like receptor 21, CpG-motif containing synthetic DNA, infectious laryngotracheitis virus, lung, chicken

## Abstract

Endosomal toll-like receptor-21 and -9 sense CpG DNA activating production of pro-inflammatory mediators with antimicrobial effects. Here, we investigated the induction of antiviral response of *in ovo* delivered CpG DNA against infectious laryngotracheitis virus (ILTV) infection. We found that *in ovo* delivered CpG DNA significantly reduces ILTV infection pre-hatch correlating with the expression of IL-1β and increase of macrophages in lungs. As assessed *in vitro*, CpG DNA stimulated avian macrophages could be a potential source of IL-1β and other pro-inflammatory mediators. Since we also found that *in ovo* CpG DNA delivery maintains increased macrophages in the lungs post-hatch, we infected the chickens on the day of hatch with ILTV. We found that *in ovo* delivered CpG DNA significantly reduces mortality and morbidity resulting from ILTV infection encountered post-hatch. Thus, CpG DNA can be a candidate innate immune stimulant worthy of further investigation for the control of ILTV infection in chickens.

## 1. Introduction

Infectious laryngotracheitis virus (ILTV) is an enveloped virus with a double stranded DNA genome and belongs to the family *Herpesviridae* and subfamily *alphaherpersvirinae* [1,2]. The ILTV causes infectious laryngotracheitis (ILT) in chickens, pheasants and peafowls worldwide [3,4], transmitted through nasal and ocular routes and results in mild to severe respiratory manifestations. The severe form of ILTV infection leads to dyspnea and bloody respiratory mucus discharge leading to mortality rate up to 70% [5]. The mild form of the ILTV infection is characterized by depression, low egg production and loss of body weights. Induction of innate immune responses characterized by the mRNA expression of pro-inflammatory cytokines and chemokines has been shown following ILTV infection in embryonic lung epithelial cells *in vitro* [6]. Protection against ILTV infection in chickens appears to be dependent on cell –mediated rather than antibody-mediated immune responses [7,8]. In addition to biosecurity measures, live-attenuated vaccines are commonly used to prevent ILT, however, increase in virulence of the vaccine strains during bird-to-bird passage is common [9,10] as has been recombination involving vaccine strains [11,12]. The limitations in current vaccine-mediated control necessitate investigations into novel control measures such as stimulation of innate immune responses in chickens.

Toll-like receptors (TLR) are a category of innate pattern recognition receptors that are responsible for recognizing pathogens via pathogen associated molecular patterns (PAMPs) at mucosal surfaces. Consequently, elicits innate immune responses linking adaptive arm of the immune system [13]. The mammalian immune system consists of TLR-1 to TLR-13 [14]. TLRs are generally conserved in vertebrates [15,16,17] except TLR-15 and TLR-21, which is unique for avian species [18,19]. In addition, the corresponding genes of mammalian TLR-8 and TLR-9 are absent in avian species [20,21,22,23], however TLR-21 is the functional counterpart of mammalian TLR-9 [19]. Similar to TLR-3, in chickens, TLR-21 is expressed in the endosomal compartment intracellularly [24] and responsible for sensing unmethylated CpG DNA [25].

CpG DNA motifs of microbial origin are known to be unmethylated and present in bacterial genome and genomes of DNA viruses at much higher frequencies than in eukaryotes [25]. Synthetic CpG DNA molecules are similar to CpG DNA originated from microbes. Depending on the structures as well as the immune responses generated, synthetic CpG DNA has been divided into three major classes, A, B and C [26]. Class A CpG DNA contains phosphodiester backbone with poly CpG DNA motifs at the center and the stimulation of cells by which leads to production of type 1 interferons (IFNs) [27,28]. Class B CpG DNA has a phosphorothioate backbone throughout and it is capable of stimulation of B cells and monocytes [28,29]. Class C CpG DNA molecules possess the properties of both class A and B CpG DNA molecules in terms of the structure and the function [30].

In mammals, it has been shown that CpG DNA could elicit protective responses in mice against bacterial infections, such as *Leishmania major*, *Chlamydia trachomatis* and *Helicobacter pylori* [31,32,33], viral infections, such as lymphocytic choriomeningitis virus (LCMV), hepatitis B virus (HBV), and poxvirus [34,35,36], and tumor conditions such as pulmonary metastases and B-cell lymphoma [37,38]. Additional to the antimicrobial and anti-tumor applications, CpG DNA is considered a potential vaccine adjuvant for human vaccines. Based on successful mice model data, human clinical studies are being conducted to investigate the efficacy of CpG DNA as an adjuvant against allergy, cancer and asthma [39,40,41], as well as hepatitis B infection [42,43].

In chickens, it has been shown that the CpG DNA is protective against bacterial infections, such as *Escherichia coli* [44,45], *Salmonella Typhimurium* [46] and *S. Enteritidis* [47], and viruses, such as avian influenza virus (AIV), *in vivo* [48] and *in vitro* [49]. In addition, protection in chickens against systemic *E. coli* and *S. Typhimurium* after hatch has been shown when CpG DNA has been delivered *in ovo* [45,46]. However, the roles CpG DNA play in the antiviral responses against avian viruses are scarce.

Furthermore, the mechanisms that lead to CpG DNA mediated antimicrobial effects in chickens have not been adequately investigated. The CpG DNA induced protection against *E. coli* and *S. Enteritidis* appears to be associated with increased functions of heterophils, including degranulation [44,50]. Recently, it has been shown that the activation of TLR-21 using CpG DNA in chicken macrophage cell line could enhance the expression of mRNA of pro-inflammatory cytokines such as IL-1β correlating with antiviral responses against AIV *in vitro* [49] and that indicates that macrophages could be another type of innate cell involved in the host responses induced by CpG DNA. It has also been recorded that the CpG DNA induced antiviral effects against IBV is associated with mRNA expression of cytokines, such as IFN-γ, IL-8 and MIP-1β, in pre-hatch embryos [51,52].

With a view of the lack of information on the CpG DNA-induced antiviral property in chickens, we hypothesized that TLR-21 ligand CpG DNA, when delivered *in ovo*, may induce antiviral responses and protection against morbidity and mortality caused by ILTV infection. We also hypothesized that the protection induced by *in ovo* CpG DNA delivery against ILTV infection is associated with expansion of macrophage populations pre- and post-hatch, as well as the production of pro-inflammatory mediators. The objectives of the study were to determine whether *in ovo* delivered CpG DNA mediate antiviral responses against ILTV infection encountered pre- and post-hatch and the protection against ILTV infection is associated with macrophage and pro-inflammatory responses in lungs of chickens.

## 2. Results

### 2.1. *In Ovo* Delivery of CpG DNA Reduces Pre-Hatch ILTV Replication in Lung of Embryos Correlating with Expansion of Macrophage Populations and Increased mRNA Expression of IL-1β in Lungs

**Figure 1:**
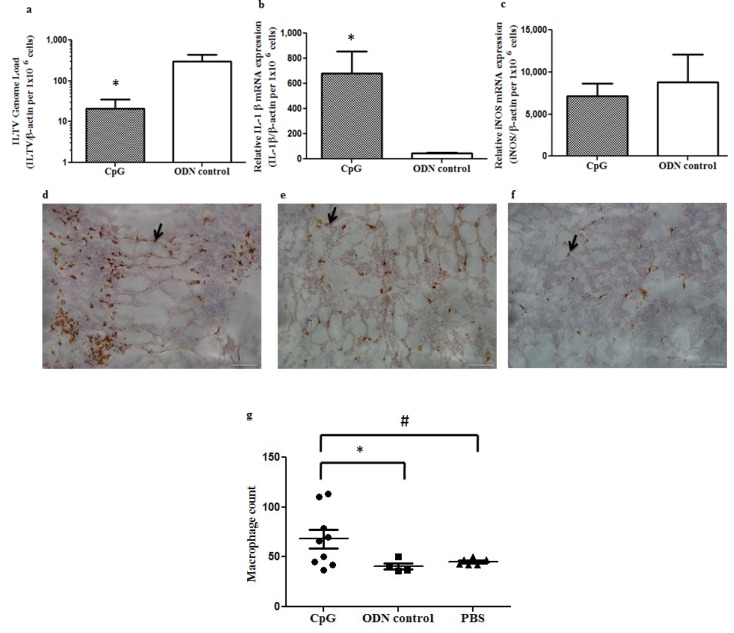
*In ovo* delivery of CpG DNA reduces ILTV replication pre-hatch in lung of chicken embryos correlating with expansion of macrophage populations and increased mRNA expression of IL-1β in lungs 24 h post treatment: (**a**) pre-hatch mRNA expression of ILTV protein kinase (PK) gene in lungs following *in ovo* CpG DNA or PBS delivery; (**b**–**c**) mRNA expression of IL-1β (**b**) and iNOS (**c**) that will be activated downstream of CpG DNA- TLR21 interaction following *in ovo* CpG DNA or PBS delivery; (**d**) macrophages in lungs pre-hatch following *in ovo* delivery of CpG DNA, non-CpG DNA and PBS 24 h post treatment (ED19). Arrows show KUL01+ macrophages * = significant at *p* ≤ 0.05 when CpG DNA treated and non-CpG DNA treated groups were compared and #= significant at *p* ≤ 0.05 when CpG DNA treated and PBS treated groups were compared.

As CpG DNA was reported to be able to induce antiviral responses *in vivo* and *in vitro* against AIV [48,49], we hypothesized that CpG DNA may be able to elicit antiviral responses against ILTV *in vivo*. For this experiment, we delivered 50 µg of CpG DNA per egg *in ovo* at ED18 with controls receiving non-CpG DNA so that treatment is available in amniotic cavity and challenged the embryos after 24 h through the same route. We found a significant decrease in ILTV mRNA expression in the lung of CpG DNA-treated embryos when compared to the controls (*p* = 0.0249, Figure 1a).

Then, we quantified the mRNA expression of the downstream molecules in the lung coinciding with the time of ILTV challenge *in ovo*, *i.e.*, 24 h post CpG DNA treatment. We found that following CpG DNA treatment IL-1β mRNA expressions in lungs were significantly increased when compared to non-CpG DNA controls (*p* = 0.0105, Figure 1b) and not the mRNA expression of iNOS (*p* ≥ 0.05, Figure 1c).

The immunohistochemistry staining of the lung tissues sampled 24 h following the *in ovo* delivery of CpG DNA, non-CpG DNA or PBS at ED18 embryos showed a significant increase in the macrophage numbers in CpG DNA treated group (Figure 1d) when compared to the non-CpG DNA treated group (*p* = 0.0448) and the PBS treated group (*p* = 0.0370). There was no significant difference between non-CpG DNA treated and PBS treated groups in terms of macrophage numbers in lung tissue pre-hatch (*p* > 0.05). 

### 2.2. Stimulation of Macrophages in Vitro with CpG DNA up Regulates mRNA Expression of Pro-Inflammatory Mediators and Increases NO Production

**Figure 2:**
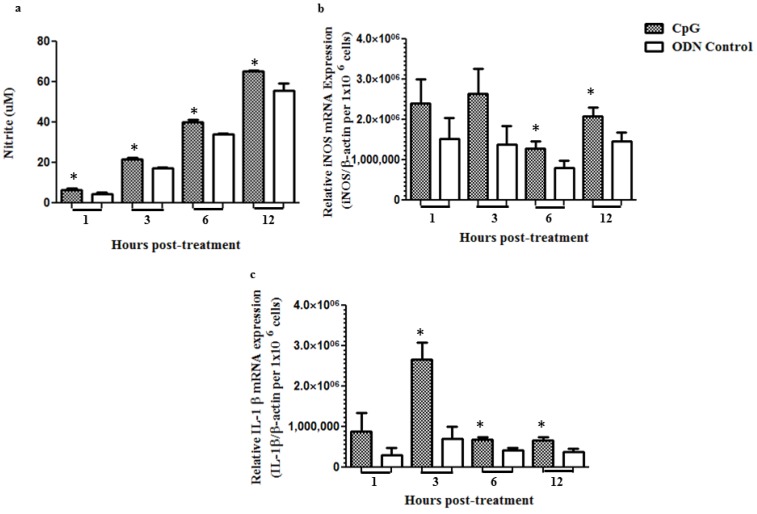
Stimulation of chicken macrophages cell line *in vitro* with CpG DNA up regulates mRNA expression of pro-inflammatory mediators and increases NO production. Avian macrophage cell line, MQ-NCSU were seeded in 6 well plates (2 × 10^6^ cells/well), cultured overnight, growth medium was removed and washed with 1 × HBSS. CpG DNA stimulation was done at 10 μM in phenol red free complete RPMI 1640 without antibiotics. Control for the CpG DNA treatment was non-CpG DNA treatment done at the same concentration. The plates were incubated for 1, 3, 6 and 12 h at 40 °C under 5% CO_2_ before collection of culture supernatants for NO (**a**) assay and cells in Trizol for RNA extraction and real-time RT-PCR for the quantification of iNOS (**b**) and IL-1β (**c**) mRNA. Each treatment was done in triplicate and the experiment was done twice under same conditions. * = significant at *p* ≤ 0.05 when student’s *t*-test was used.

Since we observed *in ovo* delivery of CpG DNA increased macrophage numbers and IL-1β mRNA expression in lungs pre-hatch, in order to clarify a potential source of IL-1β mRNA, we conducted an *in vitro* experiment stimulating an avian macrophage cell line (MQ-NCSU) with CpG DNA and non-CpG DNA. Following the stimulation, we quantified the mRNA expression of iNOS, which leads to NO production in addition to IL-1β. The NO production was found to be significantly high in MQ-NCSU cells following CpG DNA treatment when compared to that received non-CpG DNA at 1 (*p* = 0.0456), 3 (*p* = 0.0033), 6 (*p* = 0.0112) and 12 h (*p* = 0.0195) post-treatment (Figure 2a). There was a significant increase in the mRNA expression of iNOS in cells treated with CpG DNA when compared to the controls at 6 (*p* = 0.0435) and 12 h (*p* = 0.0376) post-treatment (Figure 2b). The mRNA expressions of IL-1β at 3 (*p* = 0.0100), 6 (*p* = 0.0161) and 12 h (*p* = 0.0290) post-treatment were also significantly higher than that in controls (Figure 2c).

**Figure 3:**
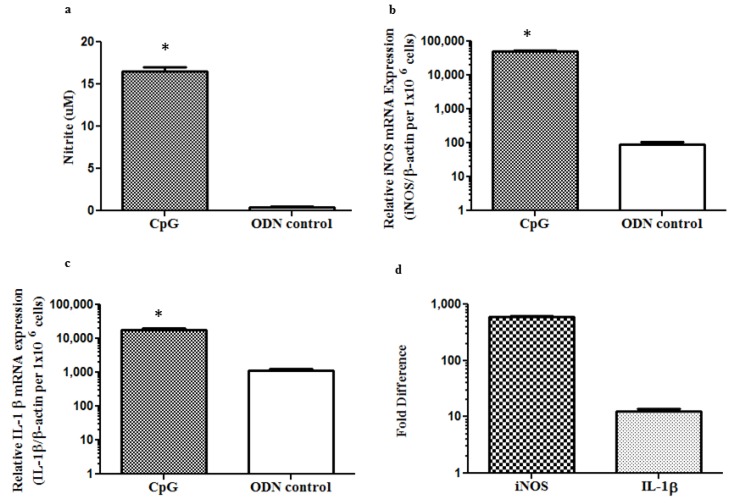
Stimulation of primary chicken macrophages *in vitro* with CpG DNA up regulates mRNA expression of pro-inflammatory mediators and increases NO production. Blood derived macrophages (1.5 × 10^6^ cells per well) were stimulated with 10 μM of CpG DNA in phenol red free complete RPMI 1640 without antibiotics and same concentration non-CpG DNA treatment was used as control for the determination of NO (**a**) and the mRNA expression of iNOS (**b**) and IL-1β (**c**) at 12 h post-stimulation; (**d**) represents the fold difference in iNOS and IL-1 β expressions by CpG DNA treated macrophage cells when compared to non-CpG DNA treated group. Differences between groups were analyzed using student’s *t*-test. * = significant at *p* ≤ 0.05 when student’s *t*-test was used.

Since we found the CpG DNA mediated increase in the expression of mRNA of iNOS and IL-1β as well as NO production by the avian macrophage cell line, MQ-NCSU, we confirmed these findings with primary chicken blood monocyte derived macrophages. We hypothesized that blood monocyte derived macrophages increase in the expression of mRNA of iNOS and IL-1β along with NO production following the CpG DNA treatment when compared to non-CpG DNA treatment. We observed a significant increase in NO production at 12 h post-treatment (*p* < 0.0001, Figure 3a). Similarly, there was a significant increase in the expressions of iNOS (*p* < 0.0001, Figure 3b) and IL-1β (*p* = 0.0003, Figure 3c) after 12 h post-treatment (*p* < 0.05, Figure 3d).

### 2.3. In Ovo Delivery of CpG DNA Expands Macrophage Numbers in Lungs Post-Hatch

**Figure 4:**
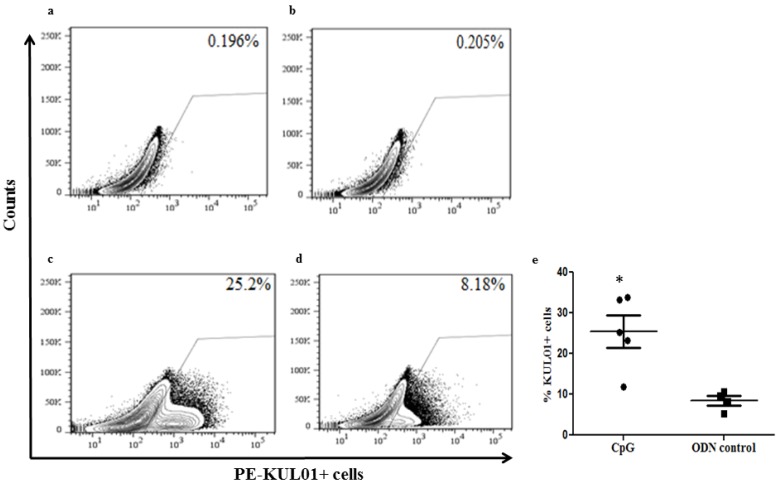
*In ovo* delivery of CpG DNA expands macrophage numbers in lungs post-hatch. (**a**–**d**) indicate the representative fluorescence-activated cell sorting diagrams showing unstained control, iso type control, percentage of KUL01+ macrophages in lungs post-hatch originated from *in ovo* CpG DNA and non-CpG DNA treated groups respectively. (**e**) illustrates the percentage of KUL01+ macrophages in lungs post-hatch originated from *in ovo* CpG DNA or non-CpG DNA delivered groups. * indicates significant differences between groups at *p* < 0.05.

Since *in ovo* delivered CpG DNA in ED18 eggs resulted macrophage population expansion in embryonic lungs after 24 h post-treatment, we hypothesized that the increased macrophage numbers following *in ovo* CpG DNA delivery may be maintained in lungs post-hatch. For the investigation of expansion of lung macrophage numbers post-hatch following *in ovo* CpG DNA delivery, we quantified the macrophages in lungs post-hatch using flow cytometry technique. We found that *in ovo* CpG DNA treatment significantly increased the macrophage numbers post-hatch when compared to that in non-CpG DNA treated controls chickens (Figure 4, *p* = 0.0041).

### 2.4. In Ovo Delivery of CpG DNA Induces Protection against ILTV Caused Morbidity and Mortality Reducing Viral Replication in the Respiratory Tract of Chickens

**Figure 5:**
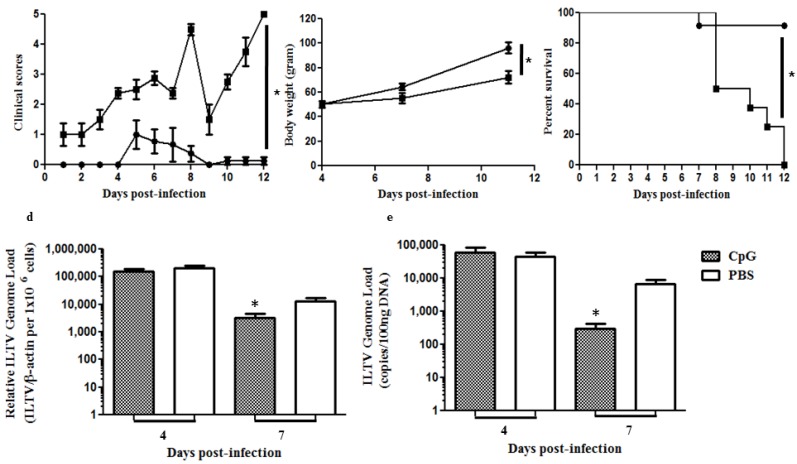
*In ovo* delivery of CpG DNA induces protection against ILTV caused morbidity and mortality reducing viral replication in the respiratory tract of chickens. SPF eggs were injected with CpG DNA or PBS *in ovo*, allowed to hatch and hatched chickens were infected with 5 × 10^4^ PFU of ILTV intratracheally. The infected chickens were observed for 12 days post-infection for development of clinical signs and determination of the end points as has been described in the materials and methods section. The chickens were also weighed (4, 7 and 11 days post-infection) and tracheal swabs (4 and 7 days post-infection) were collected for quantification of ILTV PK gene mRNA expression and genome load using real-time RT-PCR technique. (**a**) clinical scores; (**b**) bodyweights; (**c**) survival proportions, (**d**) ILTV PK gene mRNA expression as assessed in tracheal swabs using cDNA as template and (**e**) ILTV genome load in tracheal swabs using DNA as template. * indicates the significant differences between groups at *p* < 0.05.

Since we observed that *in ovo* delivered CpG DNA was capable of increasing lung macrophages post-hatch, we then hypothesized that the chickens that received *in ovo* CpG DNA leading to increased macrophages post-hatch may be able elicit protective response against ILTV infection encountered post-hatch. For this part of the study, we *in ovo* delivered either CpG DNA or PBS, allowed the eggs to hatch and then infected the hatched chickens intratracheally with ILTV on the day of hatch. We found that *in ovo* delivered CpG DNA (1) decreases clinical signs associated with post-hatch ILTV infection (*p* < 0.0001, Figure 5a); (2) protects chickens from loss of bodyweights associated with post-hatch ILTV infection (*p* = 0.021, Figure 5b); (3) decreases mortality associated with post-hatch ILTV infection (*p* < 0.0001, Figure 5c); and (4) decreases ILTV replication as assessed by mRNA expression of ILTV PK gene (*p* = 0.0276 , Figure 5d) and absolute ILTV genome load in tracheal swabs at seven days post-infection (*p* = 0.0114, Figure 5e).

### 2.5. In Ovo Delivery of CpG DNA Does Not Affect the Hatchability of Eggs

We recorded hatchability rates of 86% and 75% following *in ovo* delivery of CpG DNA and PBS, respectively.

## 3. Discussion

Our current investigations led to three major findings. Firstly, CpG DNA decreases ILTV replication in lungs pre-hatch correlating with expansion of macrophage populations and increase in mRNA expression of IL-1β in lungs. Secondly, we found that avian macrophages could be stimulated with CpG DNA *in vitro* to produce NO and increase in mRNA expression of iNOS and IL-1β genes. Finally, CpG DNA elicits protective responses against mortality and morbidity resulting from ILTV infection in chickens encountered post-hatch and this protection was associated with a reduction in ILTV replication in the lungs at a late stage of infection in the *in ovo* CpG DNA delivered chickens as well as a significant expansion of macrophage populations in lungs post-hatch.

*In ovo* route has been investigated for the delivery of CpG DNA against bacterial infections, such as *E. coli* and *S. Typhimurium*, encountered post-hatch [45,46] and found to be protective against morbidity and mortality caused by these bacterial agents. It has also been shown that *in ovo* delivered CpG DNA is efficacious as an antiviral agent against pre-hatch IBV infection [51,52]. The data we gathered in the current study show that *in ovo* delivery of CpG DNA could protect chickens from morbidity and mortality of ILTV infection encountered post-hatch implying the potential applicability of our findings for ILTV control. 

It has been shown that host responses against ILTV infection may represent initial innate responses characterized by expression of pro-inflammatory cytokine and chemokine genes [53]. It has also been observed that protective host responses against ILTV infection are associated with cell-mediated rather than antibody-mediated immune responses [7,8]. Infiltration of cells such as polymorphonuclear cells, macrophages and lymphocytes in trachea following ILTV infection has been recorded [54]. In fact, it is not clear what lung cellular components of the innate and adaptive arms are involved in the protection against ILTV infection in chickens. In the current study we observed that the *in ovo* delivery of CpG DNA related protection against morbidity and mortality induced by ILTV infection is associated with the expansion of macrophage populations in lungs pre- and post-hatch. It has been shown that CpG DNA act as a ligand for TLR-21 in chickens [19,55]. The activation of TLR-21 leads to the production of pro-inflammatory mediators [56]. In agreement with this observation, in the current study, we observed that CpG DNA treatment *in vivo* increases the mRNA expression of IL-1β in lungs pre-hatch. IL-1β is a known chemotactic factor for immune cells such as macrophages [57]. It is possible that the recruitment of macrophage populations in lungs pre- and post-hatch following *in ovo* CpG DNA delivery in our study could be due to the chemotactic function of IL-1β. In a different context, it has been recorded that CpG DNA treatment in mouse models increase recruitment of macrophages [58,59] and these records confirm our finding of increased macrophage populations in lungs following *in ovo* CpG DNA delivery. Secondly, it is also possible that CpG DNA treatment increases survival of macrophages increasing the number of macrophages accumulate in a tissue over a period of time. Our view of increased survival of macrophages leading to increased number of macrophages overtime could be supported by the finding that CpG motifs present in *Leishmania donovani* is able to inhibit programmed cell death in macrophages [60].

There are three potential circumstances to clarify the observed difference in ILTV PK gene transcripts and genome load between CpG DNA treated and controls in the present study. Firstly, there would have been a possible phagocytic role of the recruited macrophage in clearing some of the ILTV infected cells since it has been shown previously that macrophages could involve in clearing influenza virus infected cells in mice through phagocytosis [61]. Secondly, it is possible that differential NO production in macrophages between CpG DNA treated and control groups contributed to the observed difference in viral replication. To support our second view, we conducted an *in vitro* experiment using a macrophage cell line and primary macrophages and found increased production of NO by macrophages following CpG DNA treatment when compared to controls. In agreement with this explanation it has been shown previously that NO is inhibitory against avian herpes viruses including ILTV [62,63,64]. Thirdly, it is possible that differential cytokine production in macrophages between CpG DNA treated and control groups contributed to the observed difference in viral replication. It has been shown that macrophages in chickens are a known source of pro-inflammatory cytokines such as IL-1β [65] and IL-1β is known to possess direct and indirect antiviral effects that have been recorded in other host-virus models [57,66]. We also found in our study that the mRNA of IL-1β gene was up-regulated in the lungs following *in ovo* delivery of CpG DNA. We recorded that macrophages could be a source of IL-1β mRNA following stimulation with CpG DNA. Altogether, macrophages appear to be involved in the reduction of pre- and post-hatch ILTV infection in the respiratory tract following *in ovo* CpG DNA delivery.

Although we observed a reduction in the ILTV replication in lungs at a late time point in chickens originated from CpG DNA treated embryos, at four days post-infection we did not observe a difference in ILTV replication between CpG DNA treated and control chickens. Similarly, we did not see a difference in bodyweights between the CpG DNA treated and control chickens at four days post-infection but a significant difference between these two groups were observed at late time points following ILTV infection. These observations suggest that protection mediated by *in ovo* delivery of CpG DNA is not entirely depend on early innate immune responses characterized by lung macrophage population expansion and increase of expression of pro-inflammatory mediators such as NO and IL-1β. One potential mechanism may be the rapid development of adaptive immune responses in chickens that received *in ovo* CpG DNA when compared to the chickens that received PBS *in ovo*. In agreement with this view, it has been shown that CpG DNA could protect neonatal piglets from *E.coli* infection induced by enterotoxigenic strains correlating with development of adaptive immune responses, particularly mucosal antibody responses [67]. Further, CpG DNA has been shown to stimulate B cells leading to antibody production in mammals [68]. Secondly, it is possible that ILTV may be using macrophages for viral replication at the same rate of clearing ILTV infected cells using various mechanisms in the CpG DNA treated chickens at the early time points. In supporting our view, it has been shown that ILTV could utilize macrophages for it’s replication [69],

*In ovo* delivery of antigens is extensively investigated as poultry disease control methods since it is vital, due to ubiquitous nature of pathogen distribution in poultry barn environments, to employ control methods pre-hatch. Induction of host responses pre-hatch empowers the birds’ immune system at hatch and minimizes the window of susceptibility. In fact, non-pathogenic avian herpes viruses and attenuated virulent viruses have been routinely used as *in ovo* vaccines most notably for the control of Marek’s disease in chickens [70]. These *in ovo*-administered MD vaccines have proven to be efficacious in preventing morbidity and mortality induced by the causative virus. In the present study we observed that *in ovo* delivery of CpG DNA could expand macrophage populations pre-hatch as well as post-hatch strengthening the innate arm of the immune system of the hatched chickens. We also showed that the CpG DNA induced host responses such a way was capable of reducing mortality and morbidity resulted from ILTV infection encountered post-hatch. The anti-ILTV activity of CpG DNA was also found to be associated with the up regulation of mRNA of IL-1β pre-hatch. Finally, we show that *in ovo* delivery of CpG DNA found to be safe in terms of hatchability of the incubated eggs. It is imperative to investigate whether this pre-hatch stimulation of innate immune responses with CpG DNA also leads to the quick and solid antigen specific adaptive immune response against ILTV.

## 4. Materials and Methods

### 4.1. Animals

All procedures requiring the use of eggs and embryos have been approved by the University of Calgary’s Veterinary Sciences Animal Care Committee and Health Sciences Animal Care Committee. Specific Pathogen Free (SPF) eggs were purchased from Canadian Food Inspection Agency, Ottawa, Canada and incubated at the Veterinary Science Research Station (VSRS) or Health Research Innovation Center (HRIC), University of Calgary. The hatched chickens were maintained in high containment poultry isolators either at VSRS or Prion/virology animal facility at HRIC, University of Calgary.

### 4.2. Cells, Virus and TLR-Ligand

An avian macrophage cell line, MQ-NCSU, used in this study was provided by Dr. Shayan Sharif (University of Guelph, Canada). Chicken blood derived macrophages were isolated from blood as has been described previously [71]. ILTV (strain *N*-71851) was purchased from the American Type Culture Collection (ATCC, Manassas, VA, USA). CpG DNA 2007 and non-CpG DNA 2007 were purchased from Cedarlane (Burlington, ON, Canada).

### 4.3. Cell Culture

Avian macrophage, MQ-NCSU cells were cultured in LM HAHN medium which consisted of Leibovitz L-15 medium (39.5%), McCoy’s 5A medium (39.5%), chicken serum (10%), l-glutamine (1%), sodium pyruvate (1%), 100 units of penicillin and 100 µg of streptomycin per mL, fungizone (250 μg/mL), 2-mercaptoethanol (1.0 mM) (Invitrogen, Burlington, ON, Canada), tryptose phosphate broth (1%) (Sigma-Aldrich, St. Louis, MO, USA), and fetal bovine serum (8%) (Cellgro, Manassas, VA, USA) [72]. The cells were maintained at 40 °C at 5% carbon dioxide (CO_2_) in an incubator.

For the isolation of chicken peripheral blood monocyte derived macrophages jugular blood was collected in heparinized tubes and mixed with an equal volume of phosphate-buffered saline (PBS). The mixture was subjected to Ficoll gradient density centrifugation using Ficoll-Paque^TM^ PLUS (GE Healthcare Bio-Sciences AB, Uppsala, Sweden) at 400× g for 40 min at 20 °C. The cells at the interface were collected, discarding the upper plasma layer in the tube. The cells were washed in PBS and pelleted by centrifuging at 400× g for 5 min at 4 °C. This washing step was repeated three times. Finally, the cells were resuspended in RPMI-1640 with 5% chicken serum, 2% Hepes (Gibco Life Technologies, Burlington, ON, Canada), 100 units of penicillin and 100 µg of streptomycin per ml and 100 µM of L-glutamine and plated in T175 (175 cm^2^) flasks and incubated at 40 °C for 48 h.

### 4.4. Experimental Design

#### 4.4.1. The Antiviral Effect of *in Ovo* Delivered CpG DNA against Pre-Hatch ILTV Infection and Determination of Correlates of Antiviral Effect

In order to determine the antiviral effect of *in ovo* delivered CpG DNA*,* a group of ED18 eggs (*n* = 4–5) were injected with 50 µg CpG DNA in 200 µL PBS per egg and each eggs in control group (*n* = 4) were injected with 50 µg non-CPG DNA in 200 µL PBS to investigate the efficacy of *in ovo* delivered CpG motifs against pre-hatch ILTV infection. After 24 h of *in ovo* delivery, each egg was infected with 1 × 10^5^ PFU of ILTV per egg *in ovo*. The embryos were euthanized to collect lungs for RNA extraction 24 h post-infection. The experiment was done in duplicate (total of *n* = 9 in CpG DNA group and *n* = 8 in non-CpG DNA group).

In order to determine the mRNA expression of innate mediators (IL-1β and iNOS) that will be activated following *in ovo* delivery of CpG DNA, 24 h following treatment, the lungs of embryos from both CpG DNA treated group (*n* = 3) and non-CpG DNA treated group (*n* = 4) were collected for RNA extraction.

In order to determine the expansion of innate cells that will be recruited in lungs following *in ovo* delivery of CpG DNA, the lungs of embryos from both CpG DNA treated group (*n* = 9), non-CpG DNA treated group (*n* = 4) and PBS treated group (*n* = 6) were collected 24 h following treatment in optimal cutting temperature (OCT) compound (Tissue-Tek^®^, Sakura Finetek USA inc, Torrance, CA, USA) before snap freezing in dry ice. Cryosections were evaluated using immunohistochemistry technique for macrophage identification.

#### 4.4.2. Stimulation of Macrophages *in Vitro* with CpG DNA to Determine the Expression of Pro-Inflammatory Mediators

MQ-NCSU cells were propagated and cells were seeded in 6 well plates (2 × 10^6^ cells/well). After culturing the cells overnight, growth medium was removed and washed with 1 × Hanks balanced salt solution (HBSS) (Invitrogen, Burlington, ON, Canada). CpG DNA stimulation was done at 10 μM in phenol red free RPMI 1640 (Invitrogen, Burlington, ON, Canada) containing 10% FBS, 2.0 mM l-Glutamine, and no antibiotics. The plates were incubated for 1, 3, 6 and 12 h at 40 °C under 5% CO_2_ before collection of culture supernatants for NO assay and cells for RNA extraction. Each treatment was done in triplicate in two separate experiments and the results were pooled before being analyzed.

To confirm our major finding *in vitro*, we used chicken peripheral blood derived macrophages. After 24 h of culture of chicken peripheral blood derived mononuclear cells, the media was changed and after 48 h, the adhered cells were trypsinized using TrypLE^TM^ Express (Life Technologies, Burlington, ON, Canada) and counted using hemocytometer. A total of 1.5 × 10^6^ cells per well were cultured in 6-well plates and incubated for 24 h. Then the cells were treated with CpG (10 uM) prepared in phenol red free RPMI 1640 containing 10% FBS, 2.0 mM l-Glutamine, and no antibiotics and untreated wells with media alone were kept as control. After 12 h post-treatment, both supernatant and cells were collected from the wells using cell scrapper for RNA extraction. The experiment was done in triplicate.

#### 4.4.3. *In Ovo* Delivered CpG mediated Expansion of Macrophage Populations in Lungs Post-Hatch

For the evaluation of expansion of lung macrophage cell populations following *in ovo* CpG DNA delivery, ED18 embryos were injected with 50 µg CpG DNA in 200 µL PBS (*n* = 5) or non-CpG DNA control in 200 µL PBS (*n* = 4) per egg. The eggs from both groups were allowed to hatch and on the day of hatch the chickens were euthanized to collect lungs for characterization of macrophages using flow cytometry technique.

#### 4.4.4. *In Ovo* Delivery of CpG DNA Mediated Protection against ILTV Caused Morbidity and Mortality in Chickens

SPF eggs were injected with 200 µL CpG DNA (50 μg) or 200 µL PBS *in ovo*, allowed to hatch and hatched chickens (CpG DNA, *n* = 9 and PBS, *n* = 8) were infected with 5 × 10^4^ PFU of ILTV intratracheally. The infected chickens were observed daily for 12 days post-infection for development of clinical signs and determination of the end points. We considered that the chicken had reached the end point when the bird was assigned a cumulative score of 5 (ruffled feathers and huddling together = 1, droopy wings = 1, depression or head lowered with no movement = 1, mild increase in the respiratory rate = 1, increased respiratory rate with constant beak opening = 2, very severe increased respiratory rate as marked by gasping = 3 and bodyweight loss rather than gain = 1). The chickens were also weighed (4, 7 and 11 days post-infection) and tracheal swabs (4 and 7 days post-infection) were collected for quantification of ILTV PK gene mRNA expression and genome load using real-time RT-PCR and PCR techniques respectively.

#### 4.4.5. Determination of Safety of *in Ovo* Delivery of CpG DNA

We treated ED18 eggs *in ovo* with 50 μg CpG DNA (*n* = 22) and allowed to hatch along with PBS (*n* = 68) treated eggs to evaluate the hatchability.

### 4.5. Primers

The ILTV mRNA expression in lung was quantified for investigating ILTV replication using primers specific for the PK gene of ILTV as has been described previously [73] using cDNA as a template. The primers used in the relative quantification of host genes using cDNA as template were published previously [73].

### 4.6. DNA and RNA Extraction

DNA and RNA extractions from cells and respiratory tissues were carried out using Trizol reagent (Invitrogen Canada Inc., Burlington, ON, Canada) as has been described previously for other tissues [74].

### 4.7. Reverse Transcription

RNA concentration was quantified using Nanodrop 1000 spectrophotometer at 260 nm wavelength (ThermoScientific, Wilmington, DE, USA). Reverse transcription of extracted RNA (2000 ng) was carried out using 10XRT random primers (High Capacity cDNA Reverse Transcription Kit, Invitrogen Life Technologies, Carlsbad, CA, USA) according to the manufacturer’s instructions.

### 4.8. Assay for NO Production

Culture supernatants were assayed for NO production using a Griess reagent system (Promega, Madison, WI, USA) according to the manufacturer’s recommendation. The concentration of NO was quantified against a standard curve. Absorbance of the resulting colorimetric product, NO in the supernatant made in the reaction with sulfanilamide and *N*-1-naftyletylendiamin dihydrochloride was read at 548 nm.

### 4.9. Flow Cytometry Technique

Standard flow cytometry procedures were used in the experiments. Briefly, the collected lungs were chopped into small pieces and treated with 400 U/mL collagenase type I solution (Sigma-Aldrich, Oakville, ON, Canada) for 30 min at 37 °C, and neutralized by HBSS. Then the cells were layered onto equal volume of Ficoll-Paque^TM^ PLUS (GE Healthcare Bio-Sciences AB, Uppsala, Sweden) and centrifuged for 40 min at 400× g at 20 °C. The cloudy interphase layer was collected, pelleted, and washed with HBSS, and then the mononuclear cells were resuspended in complete medium. Then the cells were washed with 1% bovine serum albumin (BSA) fraction V (w/v; OmniPur, EMD, Darmstadt, Germany) made in PBS and centrifuged for 10 minutes at 1500× g (4 °C) and then suspended in 100 μL of 1:100 chicken serum (Invitrogen, Burlington, ON, Canada) (diluted in 1% BSA) for Fc blocking. Following centrifugation, the pellets were resuspended with a final concentration of 0.02 ug/µL mouse anti-chicken macrophage-monocyte phycoerythrin **(**PE) (SouthernBiotech, Birmingham, AL, USA) monoclonal antibody in the dark and incubated for 30 min on ice. The respective isotype control and unstained control were also included in the experiment. Finally, cells were washed twice with 1% BSA. The samples were analyzed with a BD LSR II (BD Biosciences, Mississauga, ON, Canada).

### 4.10. Immunohistochemistry Technique

The immunohistochemistry technique was performed as has been described previously (Abdul-Careem *et al.*, 2009). The lungs collected 24 h following *in ovo* delivery of CpG DNA, non-CpG DNA or PBS (*n* = 4–9) in order to enumerate macrophages in the lungs. The expansion of macrophage numbers was assessed quantitatively in each section by counting KUL01 positive cells with clear outlines in five highly infiltrated fields at 40× magnification.

### 4.11. Real-Time PCR Assay

All the DNA and cDNA preparations were analyzed using qPCR assays, alongside a dilution series of the plasmids used to generate the standard curve. All qPCR assays were conducted in a 96-well un-skirted, low profile PCR plate (VWR, Edmonton, AB, Canada) in a reaction volume of 20 μL. Fast SYBR^®^ Green Master Mix (Invitrogen, Burlington, ON, Canada) containing AmpliTaq^®^ Fast DNA Polymerase was used for this assay. The DNA intercalating SYBR^®^ Green dye was used for detection in a CFX96 Real-Time System C1000 Thermal Cycler (Bio-Rad Laboratories, Mississauga, ON, Canada). In addition, 5 nM of each of the gene-specific primers and 9 μL of a 1:10 dilution series of plasmid DNA, or 100 ng of cDNA extracted from each lung sample and MQ-NCSU cells, 20ng of cDNA from tracheal swabs and 50ng of cDNA from primary macrophages used in the reaction. RNAse-free water was used as the negative control. The optimum thermal cycling parameters for mRNA of IL1-β and iNOS, β actin and ILTV PK gene included pre-incubation at 95 °C for 20 s; 40 cycles of amplification/extension at 95 °C for 3 s, and 60 °C for 30 s; melting curve analysis at 95 °C for 10 s (Segment 1), 65 °C for 5 s (Segment 2) and 9 °C for 5 s (Segment 3). Fluorescent acquisition was done at 60 °C for 30 s.

### 4.12. Data Analyses

*In vitro* NO concentrations and mRNA expressions were analyzed using student’s *t*-test using GraphPad Prism 4 (GraphPad Prism Software, La Jolla, CA, USA, 2009) in order to identify differences between groups. *In vivo* data that compared ILTV genome load, ILTV PK gene mRNA expression and mRNA expression of pro-inflammatory molecules between CpG DNA treated and control chickens were also analyzed using student’s *t*-test. Before being tested, each set of data was analyzed using the Grubbs’ test (GraphPad software Inc., La Jolla, CA, USA, 2014) to identify outliers. Differences in survival between *in ovo* CpG DNA and control chickens were analyzed by chi-square (*χ*^2^) test using GraphPad Prism 4 (GraphPad Prism Software, La Jolla, CA, USA, 2009). Group differences in bodyweights was analyzed by ANOVA and clinical scores by Krusal-Wallis test using the statistical package, MINITAB^®^ release 14 (Minitab Inc., State College, PA, USA, 2004). Comparisons were considered significant at *p* ≤ 0.05.
